# Perinatal posttraumatic stress disorder as a predictor of mother-child bonding quality 8 months after childbirth: a longitudinal study

**DOI:** 10.1186/s12884-024-06570-4

**Published:** 2024-05-25

**Authors:** Maria Vega-Sanz, Ana Berastegui, Alvaro Sanchez-Lopez

**Affiliations:** 1 University Institute of Family Studies, Pontifical Comillas University, Madrid, Spain; 2 Department of Personality, Evaluation and Psychological Treatments, Complutense University of Madrid, Madrid, Spain

**Keywords:** Childbirth, P-PTSS, Bonding, Brooding, Postpartum depression

## Abstract

**Background:**

Negative childbirth experiences can be related to the onset of perinatal post-traumatic stress symptomatology (P-PTSS), which significantly impacts the mother and the infant. As a response in the face of the discomfort caused by P-PTSS, maladaptive emotion regulation strategies such as brooding can emerge, contributing to the consolidation of post-partum depressive symptoms. Ultimately, both types of symptomatology, P-PTSS and post-partum depression, can act as risk factors for developing mother-child bonding difficulties. Still, this full set of temporal paths has to date remained untested. The present longitudinal study aimed to analyze the risk factors associated with the appearance of P-PTSS after post-partum and to test a path model considering the role of P-PTSS as an indirect predictor of bonding difficulties at eight months of postpartum.

**Methods:**

An initial sample of pregnant women in the third trimester of gestation (*N* = 594) participated in a longitudinal study comprising two follow-ups at two and eight months of postpartum. The mothers completed online evaluations that included socio-demographic data and measures of psychological variables. A two-step linear regression model was performed to assess the predictive role of the variables proposed as risk factors for P-PTSS, and a path model was formulated to test the pathways of influence of P-PTSS on bonding difficulties.

**Results:**

A history of psychopathology of the mother, the presence of depression during pregnancy, the presence of medical complications in the mother, and the occurrence of traumatic birth experiences all acted as significant predictors of P-PTSS, explaining 29.5% of its variance. Furthermore, the path model tested further confirmed an indirect effect of P-PTSS, triggered by a negative childbirth experience, on subsequent bonding difficulties eight months after labor through its association with higher levels of brooding and, ultimately, postpartum depression levels. A further path showed that bonding difficulties at two months postpartum can persist at eight months postpartum due to the onset of brooding and postpartum depression symptoms.

**Conclusion:**

We identified a set of robust predictors of P-PTSS: the mother’s previous history of depression, perinatal depression during pregnancy, the presence of medical complications in the mother and the occurrence of traumatic birth experiences, which has important implications for prevention. This is particularly relevant, as P-PTSS, when triggered by a negative childbirth experience, further indirectly predicted the development of mother-child bonding difficulties through the mediation of higher use of brooding and symptoms of postpartum depression. These findings can serve as a basis for developing new longitudinal studies to further advance the understanding of perinatal mechanisms of mental health.

## Background

The childbirth experience may sometimes be associated with different uncertainties and complications, making childbirth a potentially threatening situation for some women, especially first-time mothers [1]. This can sometimes lead to experiencing childbirth as a traumatic event, being even associated with the subsequent development of perinatal post-traumatic stress symptomatology in the mother after childbirth (P-PTSS) [2]. In this study we aimed to provide an integrative analysis of the risk factors that contribute to the onset of P-PTSS and its subsequent contribution to the emergence of further crucial problems such as the emergence of mother-child bonding difficulties at post-partum.

### Traumatic childbirth and its relation to perinatal post-traumatic stress symptoms

The stress of the intervention, the perceived threat to the mother’s or baby’s life, and early separation from the baby [3] can all make childbirth a traumatic experience, impacting the mother, her baby, and the whole family [4–8]. The experience of childbirth as a traumatic event has been consistently linked to P-PTSS due to the negative psychological impact of the childbirth experience for some women [9–11]. It is estimated that around 4–16% of women may present symptoms clinically compatible with a P-PTSS after giving birth and that 30% would even meet diagnostic criteria for this disorder [12]. P-PTSS is primarily characterized by nightmares, flashbacks, irritability, guilt, and attempts to avoid thinking or talking about the birth experience [4, 8]. Some of the most prominent risk factors for the onset of P-PTSS are the presence of a previous history of depression, as well as the occurrence of depression [13], medical complications [12] and the experience of stressful life events [14] during pregnancy. The quality of medical care received during childbirth [12, 14–16], negative subjective experiences of childbirth [11–13, 15, 17–18] and medical complications in the mother [12] as in the newborn [14], such as preterm birth [11, 19], also stand out from the moment of birth itself as contributors to P-PTSS.

As it can be seen, although studies on risk factors for P-PTSS are numerous and identify multiple associated factors, these studies lack the inclusion of an integrative analysis considering this series of factors as a whole and do not take into consideration the occurrence of different risk factors that can emerge at different times (i.e., previous history, pregnancy period, time of childbirth) through longitudinal designs. Therefore, this was our first aim in this study. As a second aim, once we established the specific contribution of each of this series of known risk factors for the occurrence of P-PTSS, we aimed to establish its own contribution to further central problems of post-partum, such as the emergence of mother-child bonding difficulties, considering its influence in other individual processes of emotional management, such as maternal ruminative response styles (specifically brooding) that may facilitate the emergence of these problems.

### Ruminative response style as a strategy to regulate discomfort related to perinatal post-traumatic stress symptomatology

The cognitive model of PTSD proposed by Ehlers et al. [20] states that rumination is used as a strategy to regulate the distress generated by intrusive memories of the traumatic experience. Ruminations may comprise focusing on the traumatic event (i.e., its meaning, consequences, including intrusions, and what life would be like if the event had not occurred), with the aim of understanding it and working through it [21]. However, this form of response style would not be helpful for the person to make a coherent narrative of the trauma, rather making it challenging to integrate it [20], and thus increasing and maintaining the presence of PTSD and its severity [22].

Moreover, maintaining a rumination-focused emotion regulation style exacerbates negative emotions in the long term [20]. Specifically, depressive rumination or “brooding”, namely repetitively thinking in response to a sad mood, focusing on past problems [23], is associated with an increased vulnerability to a depressive episode [24], as well as with increases in the duration of depressive episodes [25], which makes its study particularly relevant as a possible mechanism for the maintenance of PTSD [22], but also as an origin of depressive symptomatology in the postpartum period [26].

Appropriate screening for P-PTSS risk markers may therefore have the added value of identifying mothers at increased risk for developing mood problems. However, the specific mechanisms by which the onset of P-PTSS may lead to depression and related problems in the postpartum period remain unclear, making the study of associated brooding response styles as an intervening mechanism particularly relevant.

### P-PTSS, response style, and postpartum depression

Some studies indicate that 48–55% of people diagnosed with PTSD would also meet criteria for depressive symptomatology [27]. The relationship between both pathologies could be explained by a causal relationship in which people with PTSD symptoms have a negative view of themselves, others, and the world, corresponding to the cognitive triad usually present in depression [28], which would facilitate the appearance of depressive symptomatology [29]. Specifically, P-PTSS can also act as a risk factor related to the occurrence of depressive symptomatology during the pos-partum [30]. Some studies indicate that the risk for developing postpartum depression when a woman has a single symptom of P-PTSD is 11.1 times higher than in women who do not have P-PTSS and 9.7 times higher if all PTSD criteria are met [12].

**Fig. 1:**
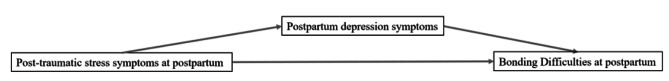
Proposed relationship between the variables P-PTSS, brooding and postpartum depression symptomatology

Looking at individual psychological processes, the relationship and high comorbidity between both symptomologies could be explained through the presence of common factors such as negative thoughts, which are symptoms in both clinical conditions [11]. In that way, brooding has been identified as a further predictor of postpartum depressive symptomatology during the puerperium [26]. It could therefore be argued that P-PTSS may facilitate postpartum depressive symptomatology through a higher use of ruminative brooding as a maladaptive emotion regulation strategy to cope with distress (see Fig. 1).

Importantly, several authors point out how the presence of these two types of symptomatology in the mother during the postpartum period can significantly influence the quality of mother-child bonding, which is a fundamental aspect for the correct development of the baby [31–33]. In this study, we thus finally aimed to consider how P-PTSS and post-partum depression, and through which paths, contribute to mother-child bonding difficulties across the post-partum period.

### Mother-child bonding difficulties

Mother-child bonding comprises the mother’s mental representation of her child and her caring behaviors towards her child [34]. The consequences of difficulties in mother-child bonding are problems in emotional self-regulation, problems in stress regulation and social adaptation, alterations in the infant temperament, and difficulties in cognitive and social development from the age of three months [31].

Symptomatology of P-PTSS, brooding [33], as well as postpartum depression [31], have all been found to be related to a poorer quality of mother-child bonding across separate studies. In the case of P-PTSS, mothers who experience P-PTSS tend to have less experiences of positive emotions toward the infant [35], difficulties in caregiving tasks, and problems establishing quality bonding [36–37]. In addition, mothers with P-PTSS also frequently experience emotions of anger towards the infant [36] and a decrease in their perceived self-efficacy [3]. It has also been reported in the literature that, in these cases, the newborn may act as a reminder of the traumatic event, producing feelings of rejection of the mother towards her child in the initial moment [13, 38–39] Moreover, the experience of P-PTSS by the mother acts as a risk factor for her child to exhibit poorer emotion regulation at 6 months [39], lower cognitive development at 17 months [40], and worse socio-emotional development at 2 years [41].

Some previous research on P-PTSS and its relationship with bonding quality suggests that this relationship might be mediated by the presence of postpartum depression symptoms [40, 41]. verall PTSD symptomatology directly impacted bonding quality but also indirectly through depressive symptomatology [11]. The relationship and high comorbidity between both symptomologies could be explained through the presence of common factors such as negative thoughts, which are symptoms in both clinical conditions [11]. Additionally, the relationship between P-PTSS and the presence of depressive symptoms in the postpartum period could be mediated using brooding as a maladaptive emotion regulation strategy to cope with distress [26]. Moreover, brooding has been related to a poorer quality of mother-child bonding in the postpartum period [33] because depletes the mother’s cognitive resources, making it difficult for her to manage the new cohabitation with a newborn, and limiting her ability to respond to the baby’s needs in a responsive, rapid, and congruent manner [33]. It that way, a higher use of brooding may facilitate postpartum depressive symptomatology. When the mother presents depressive symptomatology during the postpartum period, she tends not to bond adequately with her child, which may cause her to emotionally distance herself from her child, experiencing fewer positive feelings towards her baby, isolating herself from the baby’s emotional demands and attending only to his/her basic needs [32]. Furthermore, it has been found that children of mothers with postpartum depression exhibit poorer cognitive and socioemotional functioning in preschool and middle childhood [42–43].

**Fig. 2:**
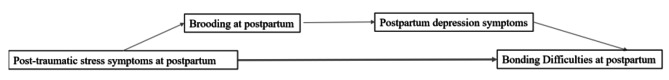
Proposed path model

Overall, it is crucial to continue expanding research in this area [44] Specifically, it is necessary to clarify the possible influence of P-PTSS on lower-quality bonding during the postpartum period through its indirect impact on the use of brooding and associated depressive symptomatology. Hence, the second aim of this study was to formulate and test an empirically informed and integrative path model, with this being particularly relevant to know the processes through which P-PTSS, after childbirth, may ultimately lead to the appearance of bonding problems at eight months of postpartum, by the hypothetical mediation of brooding and postpartum depression symptoms (see Fig. 2 below).

Finally, the experience of childbirth, the birth itself, should be seen as an event that takes place in each socio-cultural and health care environment, which influences the experience itself. In terms of the socio-cultural context, in Spain there are different healthcare services: public health service, private medical insurance and entirely private healthcare. The latter two options offer greater flexibility in choosing the hospital and doctor. Most deliveries take place in public or private hospitals, with the option of using birthing centers being very uncommon, and home births being extremely rare. The delivery can be either vaginal or by cesarean section, depending on the circumstances and the preferences of the healthcare professionals. During labor, women are attended to by midwives and obstetricians, but the figure of the doula is not yet established. The presence of a companion is usually allowed, except for cesarean deliveries, which varies by hospital. Options for pain control are offered, with pharmacological methods being the most prevalent nowadays, especially the use of epidurals.

In terms of the health context, the health crisis caused by the COVID-19 pandemic was still occurring at the time of the present study. Women in this study gave birth between November 2020 and May 2021. During these months, the COVID-19 pandemic in Spain was at its peak, triggering the implementation of many new protocols in the hospital context. Despite the challenges posed by the pandemic, a combination of childbirth options was maintained, and the presence of a companion continued to be valued, although temporary restrictions could vary depending on hospital policies and health conditions. Similarly, despite the limited number of community services for parents during the postpartum period in Spain, efforts were made to provide telephone and online follow-ups to address potential difficulties in the early stages.

### Study aims

In this study, we considered women’s negative childbirth experiences and how they may sometimes lead to the development of Perinatal Post Traumatic Stress symptoms (P-PTSS), as well as the specific paths of contribution of this problem to subsequent mother-child bonding difficulties across the postpartum. Therefore, two main aims were defined, and were addressed through an extensive longitudinal study with multiple time assessments across the pregnancy and post-partum period. First, we conducted a detailed analysis of the risk factors occurring before and during pregnancy, and at childbirth, identified by previous empirical research [12–18], that can predict a higher presence of P-PTSS after childbirth. Specifically, to address this first aim, we conducted an integrative study of predictors for P-PTSS that have been separately identified as risk factor in the previous literature, including pre-pregnancy (previous history of depression; 13), during pregnancy (presence of perinatal depression symptoms, generalized anxiety symptoms, the experience of stressful life events and medical complications; 12–14), as well as childbirth factors (quality of medical care received, birth experience and infant complications during childbirth; 12–18). To study the relationship between all these factors and P-PTSS, a first section of the longitudinal design was formulated to integrate them and consider their occurrence at different times (pre-pregnancy, during-pregnancy, and childbirth-related predictors of P-PTSS at eight months of postpartum).

As for the second aim, we formulated a path model to test the hypothesis that P-PTSS has an indirect predictive role in bonding difficulties at eight months of postpartum through its direct influence on brooding and associated post-partum depressive symptomatology (see Fig. 2 for more details). Thus, based on the reviewed literature, it was expected that a higher presence of P-PTSS after childbirth would predict subsequent higher levels of brooding [20–22], which would be associated with higher postpartum depressive symptomatology levels [3, 12], and that in turn, these problems would be associated with higher increases of mother-child bonding difficulties across the postpartum period [31, 33].

## Methods

### Design and participants

A longitudinal study with three data collection phases was designed. A non-discriminatory exponential chain demonstration (i.e., snowball sampling technique) was performed to recruit the sample. The inclusion criteria for the study were being a woman over 18 years of age and in the third trimester of pregnancy. The first evaluation (T1) comprised recruiting an initial sample of women of *N* = 594 in their third trimester of pregnancy. This phase was conducted from November 2020 to February 2021. The mean week of gestation in women recruited at T1 was 33.37 (SD = 3.77). The second evaluation (T2) consisted of reassessing all T1 participants two months after childbirth. A sub-sample of *N* = 326 was obtained from T1. This phase was conducted between January and July 2021. The average week of gestation at which participants went into labour was 39.33 (SD = 1.57). Finally, in the third evaluation (T3), the longitudinal follow-up of T2 participants continued. This T3 evaluation was completed eight months after giving birth and six months after the previous assessment at T2. This final sample was the one used in this study as all T3 participants had completed the longitudinal follow-up of all three phases. This phase was conducted between July 2021 and January 2022.

The mean age of the final sample was 32.16 (SD = 4.18), 74% of the participants had completed university studies, 95% had a couple or were married at the time of the first evaluation. The average number of children of the participants was 0.33 (SD = 0.55), and 68% followed up on their pregnancy by the public health system.

The 26% of the women reported psychotherapy during the first eight months postpartum and 6.8% reported using anxiolytics during that time. Descriptive socio-demographic data are shown in Table 1. All participating women signed informed consent. The Ethics Committees of Universidad Pontificia Comillas approved the study protocol .

**Table 1:** Descriptive data of socio-demographic and psychological impact variables

Variable	Total Sample (*N* = 150)
	%
Stressful life event during pregnancy (T1)	
No	84
Yes	16
Complications during pregnancy (T1)	
No	81
Yes	19
Type of delivery (T2)	
Vaginal	76
Cesarean section	0.7
Cesarean section with previous labor	15.8
Scheduled cesarean section	6.2
Home birth	1.4
Complications in the mother during childbirth (T2)	
No	71.2
Yes	28.8
Complications in the baby during childbirth (T2)	
No	71.9
Yes	28.1

Note. T1: third trimester of pregnancy; T2: two months postpartum

### Instruments

Data were collected through online evaluation surveys using Google Forms. First, the surveys included questions on socio-demographic data: age, type of medical care, marital status, educational level, and type of delivery. The surveys also included questions testing the psychological impact of variables such as medical complications for the mother during pregnancy (i.e., ‘Did you have any medical complications during your pregnancy?‘) and during childbirth (i.e., ‘Did you have any medical complications during childbirth?‘), and the baby afterward during delivery (i.e., ‘Did your baby have any medical complications during childbirth?‘) all based on a yes/no answer option. If mothers answered ‘yes’ to any of these questions, they were asked another question about the type of complication they experienced (e.g., ‘Could you indicate what type of complication/s it was/were?‘). The quality of medical care received and the type of birth experience (i.e., whether distressing or even traumatic) was assessed through single questions formulated by the researchers (i.e., “In relation to the medical care received during the birth, please indicate how you experienced it: satisfactory, adequate, negative or threatening”; “In relation to your birth experience, please indicate whether you experienced it as a satisfactory birth, a difficult but satisfactory birth or a traumatic birth”).

Secondly, the following standardized questionnaires were used to assess risk factors for P-PTSS, as well as the variables used to test the relationship between P-PTSS and subsequent brooding, depression and mother-child bonding.

**Perinatal posttraumatic stress symptoms**. PCL-5, PTSD Checklist for DMS-5. The Post-traumatic Stress Disorder Symptom Checklist [45] comprises 20 items and a Likert-type response scale (5 response options). It was specified that mothers should complete this questionnaire based on their birth experience. The internal consistency in this study at eight months postpartum was excellent α = 0.93. This questionnaire was administered at T3.

**Previous history of depression**. The Centre for Epidemiological Studies Depression Scale, CESD-8 [46], is an 8-item scale that can be used to detect the occurrence of depressive episodes at different periods. This study assessed the degree of depression levels experienced in the worst period of the previous life identified by the participants before the third semester of pregnancy. The items are rated on a 4-point Likert scale. The scale shows a Cronbach’s alpha of 0.82 in Spanish validation [47]. The internal consistency in this study was good α = 0.89. A cut-off score ≥ 9 was used to indicate the existence of a previous history of depression. Participants were divided into two groups according to the above cut-off for the total score obtained on this questionnaire. Those who scored below the cut-off point were assigned to group 0 (i.e., no previous history of depression), and those who scored at or above the cut-off point were assigned to group 1 (i.e., presence of a prior history of depression). This questionnaire was administered at T1.

**Stressful life events.** Participants were asked to indicate whether they had experienced any stressful life events from the following pre-specified list of life stressors [48] during their pregnancy: separation or divorce, death in the family, job loss, domestic violence, or unwanted pregnancy. Participants who did not select any stressful life events were assigned to group 0 (i.e., no significant stressors occurred), and those who did were assigned to group 1 (i.e., significant stressors occurred). This questionnaire was administered at T1.

**General anxiety.** The Generalised Anxiety Scale, GAD-7 [49], is a 7-item scale rated on a 4-point Likert scale. A cut-off score ≥ 10 was used to identify clinical levels of anxious symptomatology at the time of assessment [49]. This scale shows a Cronbach’s alpha of 0.93 in its Spanish validation [50]. The internal consistency in this study, in the third trimester of pregnancy, was good α = 0.87. This questionnaire was administered at T1.

**Perinatal and post-natal depression**: The Edinburgh Postnatal Depression Scale, EDPS [51], has been validated in the perinatal and postnatal stages. It is a 10-item scale, and items are rated on a 4-point Likert scale. The cut-off score ≥ 13 indicates a high risk of depression during pregnancy and ≥ ten postpartum. The internal consistency of the Spanish adaptation of the instrument was 0.91 [52]. Internal consistency in this study, in the third trimester of pregnancy, was good, α = 0.86 and α = 0.88 at eight months postpartum. This questionnaire was administered at T1, T2, and T3.

**Ruminative Response.** The Ruminative Response Scale, RRS [53], was used to assess individual styles of ruminative response, differentiating between the use of reflection (reflective rumination) and brooding (depressive rumination). It is a 22-item scale. The items are rated on a 4-point Likert scale. The Spanish adaptation shows a Cronbach’s alpha of 0.93 for the full scale [54]. At eight months postpartum, the internal consistency in this study was excellent α = 0.94 for the total scale, and good α = 0.82 for the brooding subscale. This questionnaire was administered at T3.

**Postpartum bonding difficulties.** The Postpartum Bonding Questionnaire [55] was used to detect the presence of bonding difficulties. It provides a total score of the quality of bonding. It comprises 25 items with a Likert-type response scale (5 response options). The adaptation to Spanish shows a Cronbach’s alpha of 0.90 on the full scale. The internal consistency in this study, at the two postpartum assessment periods was good, α = 0.83, at two postpartum months and α = 0.86 at eight postpartum months. This questionnaire was administered at T2 and T3.

Complementarily, further measures of other variables not relevant to the aims of this study were also collected in the study. The full set of measures relevant to this study, collected at each assessment time, are summarized in Table 2.

### Statistical analyses

Statistical analyses were performed using IBM SPSS Statistics version 22 software. Initially, descriptive analyses of socio-demographic data and psychological measures of the participants were performed. In addition, a preliminary analysis with t-tests for independent samples was carried out on the sample’s representativeness.

Subsequently, Pearson bivariate correlation analyses were carried out on the association between the risk factors proposed and P-PTSS (i.e., aim 1) and the principal variables of the subsequent path model (i.e., aim 2: perinatal post-traumatic stress symptom score → brooding → depressive symptomatology → bonding difficulties). Moreover, t-tests for independent samples were carried out for differences in the dimensional variables by other risk dichotomous variables. Following Cohen’s [56] criteria, the following magnitudes will be followed to interpret the results: between +/- 0.10 and +/- 0.29 low; between +/- 0.30 and +/- 0.49 medium or moderate; between +/- 0.50 and +/- 1.0 high correlation.

As for the first aim, to determine the percentage of explained variance of the dependent variable (P-PTSS at T3) by the variables considered as predictors, a linear regression analysis was performed in three steps, considering the contribution of antenatal variables (T1; step 1) pregnancy variables (T1; step 2) and partum variables (T2; step 3).

**Fig. 3:**
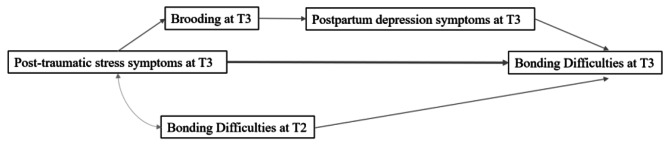
Initial proposed path model Note. T2 = two months of postpartum; T3 = eight months of postpartum

As for the second aim, we tested the initial hypothesized path model (see Fig. 2) using a structural equation that included the complete set of significantly correlated variables. In that model, P-PTSS (T3) acted as an exogenous variable, predicting bonding difficulties directly (T3) and indirectly through the influence of brooding (T3) (i.e., mediator 1) and postpartum depressive symptomatology (T3) (i.e., mediator 3). For this purpose, bonding difficulties at two months postpartum (T2) were controlled (i.e., regressed on bonding difficulties at eight months postpartum, T2), which allowed to establish the change in bonding that was solely due to the proposed predictor variables in the model. The estimation of standardized parameters of the path model followed the full information maximum likelihood (FIML) estimation method. Adjustment of our model we tested using standard criteria [57]: (a) χ2: non-significant value; (b) χ2 / gl: values lower than 2;c) comparative fit index and Tucker-Lewis index, a value ≥ 0.95 indicates a good fit ; d) root mean square error of approximation, a value ≤ 0.05 indicates a good fit; e) standardized root mean square, a smaller value indicates a better fit between the observed data and the tested model; and f) Akaike information criterion, a lower value indicates the preference for selecting a model when compared to another model [58–59]. Moreover, we used the Mardia coefficient for assessing multivariate normality (a value ≤ 70 indicates the possibility to assume multivariate normality) [60]. Hypothesized mediation pathways (i.e., P-PTSS → brooding → depressive symptomatology → bonding difficulties) with the variable of previous levels of bonding difficulties (T2) being a controlled, providing a measure of temporal change of bonding difficulties (Fig. 3), were tested via the estimation of indirect effects within the full path model. The model was then reformulated following modification indices and considering the predictive role of childbirth complications at T2 in P-PPTSS levels as T3 as the predictor (see the Results section for full details on this final model). The final model’s adjustment and its mediational pathways on T2-T3 changes in bonding difficulties were tested following the same criteria and steps as detailed above. These structural equation models and the analyses of the resulting path analyses were conducted using AMOS v18.0 (SPSS).

## Results

Descriptive data of socio-demographic data and psychological impact variables of the participants in the study are shown in Table 1 above. Descriptive data of dichotomous psychological measures, and further mean and standard deviations of dimensional psychological measurements are depicted in Table 2 below.

**Table 2:** Descriptive data of dichotomous psychological measures, and further mean and standard deviations of dimensional psychological measurements

Variables	(%)	T1, M (SD)	T2, M (SD)	T3, M (SD)
Previous depression (%)				
No	41.8			
Yes	58.2			
Perinatal/Postpartum depression		8.36 (*5.31*)	8.14 (*5.40*)	8.49 (*5.51*)
General anxiety		7.16 (*4.73*)	7.01 (5.02)	7.53 (*5.33*)
Brooding		4.08 (*3.39*)	3.64 (*3.28*)	4.08 (*3.39*)
Bonding difficulties			12.75 (*8.18*)	17.50 (*7.70*)
Posttraumatic stress symptom level				9.42 (*11.30*)

Note. M = Mean; SD: Standard Deviation; T1: third trimester of pregnancy; T2: two postpartum months; T3: eight postpartum months

### Representativeness of the sample

For psychological measures that were collected multiple times, no significant differences were found in the variables collected in T1 between completers and non-completers at T2 (perinatal depression t= -0.998; bonding difficulties t= -0.495; brooding t = 0.650; all p’s > 0.05) and completers and non-completers at T3 (perinatal depression t= -0.335; bonding difficulties t= -0.501; brooding t = 1.407; all p’s > 0.05). No differences were neither found in variables collected in T2 between completers and non-completers at T3 (postpartum depression t= -0.402; bonding difficulties t= -0.870; brooding t= -0.845; all p’s > 0.05).

### Risk factors of post-traumatic stress symptoms at eight months of postpartum

#### Bivariate correlations and T-tests for independent samples

Bivariate correlation analyses showed significant correlations between post-traumatic stress symptoms and risk factors assessed to test the first aim. Specifically, P-PTSS (T3) were significantly moderate and positively related to perinatal depression (T1; *r* = 0.448; *p* = 0.001), moderate and positively related to general anxiety (T1; *r* = 0.388; *p* = 0.001), low and positively related to quality of care received (T2; *r* = 0.196; *p* = 0.018) and low and positively related to childbirth experience (T2; *r* = 0.277; *p* = 0.001).

The t-tests for independent samples indicated that women with a history of depression (T1) had, in comparison to those without a previous depression episode, higher P-PTSS (T3), t(148) = − 4.897; *p* = 0.001. There were no statistically significant differences in P-PTSS (T3) as a function of differences in the experience of a stressful life event during pregnancy (T1), t(36.815) = 0.748;*p* = 0.459, the presence of complications in the mother during the pregnancy (T1), t (148) = 1.678;*p* = 0.095, and during the childbirth (T2), t (148) = 0.460;*p* = 0.646,or the presence of complications in the baby during childbirth(T2), t (77.300) = − 0.532;*p* = 0.596.

#### Main analyses (Aim 1): Linear regression model considering three-step predictors of P-PSTD

The linear regression model was composed of three steps. In the first step [1] pre-pregnancy predictor variables were entered: previous history of depression (assessed retrospectively at T1). In the second step [2], psychological predictor variables present during the third trimester of pregnancy: perinatal depressive symptomatology, generalized anxiety symptomatology, the experience of stressful life events, and complications during pregnancy (T1) were entered (assessed at T1). In the third step [3], predictor variables specific to the childbirth were entered in the equation: quality of medical care received, birth experience, and complications in the baby after delivery (assessed retrospectively at T2, two months after labor). The outcome variable of the model was P-PTSS, assessed eight months after childbirth (T3). The results of the linear regression model are shown in Table 3.

**Table 3:** Linear regression model of the dependent variable post-traumatic stress symptoms

Predictor variables	*R*	*R* ^2^	Adjusted R^2^	SE	Sig.
Before Pregnancy (1)	0.378	0.143	0.137	10.503	0.001**
Pregnancy (2)	0.524	0.274	0.248	9.801	0.001**
Childbirth (3)	0.582	0.339	0.295	9.491	0.001**
Predictor variables	β		t		Sig.
History of previous depression (T1)	0.203		2.606		0.010**
Perinatal depression (T1)	0.257		2.357		0.020*
General anxiety (T1)	0.121		1.129		0.261
Stressful life events during pregnancy (T1)	-0.104		-1.464		0.145
Pregnancy complications (T1)	-0.129		-1.813		0,072
Quality of care received (T2)	0.075		0.889		0.376
Childbirth experience (T2)	0.202		2.405		0.018*
Complications in the mother during childbirth (T2)	-0.154		-2.094		0.038*
Complications in the baby during childbirth (T2)	0.004		0.051		0.960

Note. 1: History of previous depression. 2: Perinatal depression, general anxiety, stressful life events during pregnancy, pregnancy complications. 3: Quality of care received, childbirth experience and complications in the baby during childbirth; T1: third trimester of pregnancy; T2: two postpartum months; T8: eight postpartum months. * Regression coefficient significant at *p* < 0.05 **Regression coefficient significant at *p* < 0.01

As can be seen in the table, 29.5% of the variance in P-PTSS (T3) at eight months postpartum was explained by the model. The significant positive predictive power of the a previous history of depression (Step 1) explained 13.7% of the variance in P-PTSS in the eighth month of postpartum. A further 11.1% of variance in P-PTSS was accounted by predictors referred to the pregnancy period (Step 2), from which perinatal depression was the only significant rpedictor. Finally, a further 4.7% of the variance in P-PTSS was predicted by predictors referred to the childbirth (Step 3), among which medical complications in the mother during childbirth and the birth experience as traumatic acted as the significant predictors. Thus, more threatening, and traumatic women’s appraisal of their birth experience accounted for greater levels of P-PTSS at eight months of postpartum.

### Predictive role of post-traumatic stress symptoms at eight months of postpartum on bonding difficulties

#### Bivariate correlations

Preliminary bivariate correlation analyses supported significant correlations between the main variables under study (aim 2). All variables were significantly related. Postpartum depression (T3) was significantly moderate and positively related to brooding (T3), *r* = 0.670; *p* = 0.001, bonding difficulties (T3), *r* = 0.517; *p* = 0.001, and P-PTSS (T3), *r* = 0.641; *p* = 0.001. Brooding was significantly moderate and positively related to bonding difficulties at T3, *r* = 0.526; *p* = 0.001, and P-PTSS at T3, *r* = 0.613; *p* = 0.001. Finally, P-PTSS was significantly moderate and positively related to bonding difficulties at T3, *r* = 0.482; *p* = 0.001.

#### Main analyses (Aim 2): path model of P-PTSS as an indirect predictor of mother-child bonding

Based on the previous bivariate correlation analysis we tested an equation model where bonding difficulties at eight months of postpartum (T3) variables were predicted by P-PTSS (at eight months of postpartum; T3) directly and/or indirectly through the use of brooding (T3) as emotional regulation strategy and the development of postpartum depressive symptoms (T3), with the variable of previous levels of bonding difficulties (T2) being a controlled, providing a measure of temporal change of bonding difficulties. All the goodness-of-fit indices are shown in Table 4.

**Table 4:** Goodness of fit indices for the tested models

	Chi-square (df)	*P* value	Χ^2^/df	CFI	TLI	RMSEA (90% CI)	SRMR	AIC
*Model 1*	*7.473* [2]	*0.024*	*3.736*	*0.981*	*0.906*	*0.137 (0.043/0.248)*	*0.049*	*320.139*
*Model 1R*	*1.850* [6]	*0.933*	*0.308*	*1*	*1.035*	*0.000 (0.000/0.029)*	*0.015*	*336.700*

Note. CFI: comparative fit index, TLI: Tucker-Lewis index, RMSEA: root mean square error of approximation, SRMR: standardized root mean square, AIC: Akaike information criterion; Model 1: Initial Model; Model 1 R: initial model respecified

**Fig. 4:**
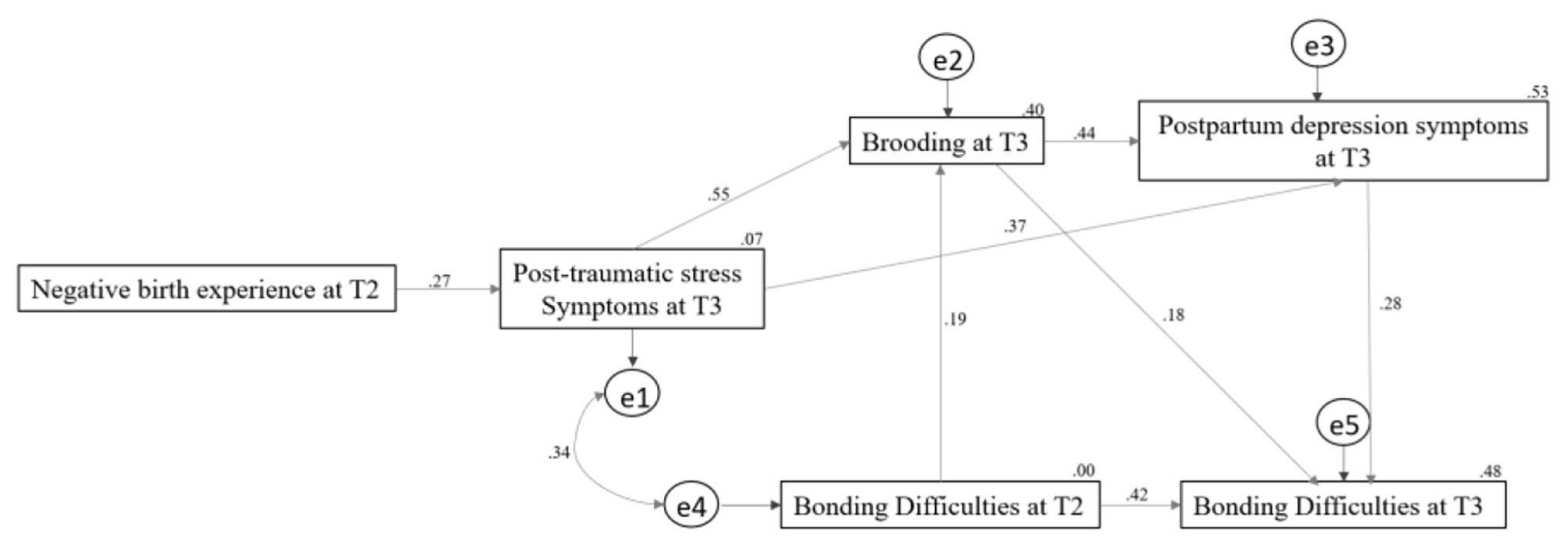
The respecified model (Model 1R) with standardized regression weights Note. T2: 2 months postpartum. T3: 8 months postpartum

As shown in Table 4, the goodness-of-fit indices for the initial hypothesized path model were not good. Moreover, the Mardia coefficient yielded a value of 15.94 which is far for the critical value (± 5). Since the fit of our initial model (Model 1) was poor, re-specification was carried out following Wald and Lagrange multiplier tests [61]. All paths with nonsignificant *P* values were removed consecutively. Only the path P-PTSS to bonding difficulties, both at 8 months of postpartum was removed. The variable ‘birth experience,’ assessed at 2 months postpartum (T2), was further introduced as a predictor of the onset of P-PTSS at 8 months postpartum (T3), and an additional path was included in the model, through which bonding difficulties at 2 months of postpartum predicted brooding at 8 months of postpartum (Fig. 4). The final respecified model (Model 1R) showed a very good fit in all the indices (Table 4). Despite the Mardia coefficient remaining slightly elevated at 13.67, it improved compared to Model 1, and it would be appropriate to use maximum likelihood estimation as it did not exceed the value of 70 [60].

Indirect effects were tested using bias-corrected bootstrap estimations (2000 bootstrap samples with 95% CI). First, the hypothesized path was significant, with an indirect effect of P-PTSS triggered by a negative birth experience at T2 on bonding difficulties’ changes from T2 to T3, through individual differences in P-PTSS in ruminative brooding, subsequently leading to higher postpartum depression symptom levels and ultimately resulting in lower bonding (*p* = 0.001; SE = 1.375; 95% CI 3.759 to 8.281), supporting the hypothesized path. Additionally, a further indirect effect of P-PTSS after a negative birth experience on bonding difficulties, exclusively through individual differences in brooding after controlling for depression levels was also statistically significant (*p* = 0.001; SE = 1.388; 95% CI 3.053 to 7.606). There was also a significant indirect effect of P-PTSS after a negative birth experience on bonding difficulties, exclusively through postpartum depression symptoms after controlling for brooding levels (*p* = 0.001; SE = 1.370; 95% CI 3.062 to 7.575). Furthermore, the indirect effect of bonding difficulties at two months (T2) on bonding difficulties at eight months postpartum (T3) through individual differences in ruminative brooding, subsequently leading to higher postpartum depression symptom levels and ultimately resulting in lower bonding, was significant (*p* = 0.001; SE = 0.161; 95% CI 0.917 to 1.449). Additionally, the significant indirect effect of low-quality bonding (T2) on bonding difficulties (T3) through individual differences in brooding, after controlling for depression levels, was also statistically significant (*p* = 0.015; SE = 0.192; 95% CI 0.182 to 0.821).

## Discussion

The present study considers the type of birth experience for the mother as a bridging event between pregnancy and postpartum psychological experiences, whose relevance is of great importance in the postpartum period, not only for the psychological well-being of the mother but also for the bond with her child [4–8]. Sometimes childbirth is not experienced as a positive but as a negative or even traumatic event [12], which can lead to P-PTSS [9–10]. Previous research has been focused on comprehensively studying the risk factors that may influence P-PTSS development after childbirth. Yet, these previous studies did not typically consider all possible predictors in an integrative manner, neither used different temporal assessments of relevant risk factors at different relevant periods (before and during pregnancy, and at childbirth). The first aim of the present study was thus to assess the risk factors identified in previous literature for the development of P-PTSS, but integrating all the factors identified in different studies into the model, while also taking into account their occurrence at different times (i.e., prior to pregnancy, pregnancy period, time of childbirth), through a rigorous and extensive longitudinal design.

Results indicated that the presence of an history of depression before pregnancy as well as the higher presence of depressive symptoms during the third trimester of gestation both stood out as significant predictors of P-PTSS eight months after childbirth. These results align with evidence from previous studies [13] and show the impact of the mother’s life history and current psychological well-being during pregnancy on her childbirth experience. Moreover, experiencing medical complications during childbirth by the mother, as well as perceiving the childbirth experience as threatening or traumatic, were both identified as additional significant risk factors for the subsequent development of P-PTSS. Thus, the more complex and traumatic the mothers’ childbirth experience, the greater the probability of developing P-PTSS. These results align with evidence from previous research on this issue [12, 14–15, 17], while being obtained in an integrative model that consider all these variables at different temporal stages of pregnancy and childbirth.

In contrast, generalized anxiety symptomatology during pregnancy, the experience of stressful life events and complications during pregnancy, the quality of medical care received, and the complications in the baby during birth were not found to be significant predictors of P-PTSS above and beyond the mentioned main risk factors. These results apparently contrast to the ones from other studies [12–15]. Nonetheless, the absence of predictive power of these other variables could be due to the lack of representativeness and variability of these variables in the present sample. The participants’ mean anxiety level in this study was below the cut-off point on the generalized anxiety symptomatology questionnaire during pregnancy, 84% did not report to have experienced stressful life events, and 81% did not report to have experienced medical complications during pregnancy. Similarly, 72% did no have medical complications after delivery. Finally, the medical quality variable could not be a predictor due to limitations in the assessment method, as it was only assessed through a single question that did not show sufficient sensitivity.

Overall, using an integrative and extensive longitudinal design, the present study allowed to determine the main risk factors contributing to higher levels of P-PTSS. This seems essential, as this problematic has been identified as a core source of dysfunction for some mothers itself but also a further predictor of additional central problems at postpartum, such as the appearance of difficulties in mother-child bonding. Yet, the specific paths for this influence remained to date unclear.

Thus, the second aim of the study was establishing the indirect impact of P-PTSS symptomatology on mothers’ bonding with the newborn through other identified regulatory and mental health issues emerging during postpartum (i.e., brooding, postpartum depression). The initial path model formulated (see Fig. 3 for more details) did not show good fit indices, so we followed modification indices and introduced the variable ‘birth experience’ into the model as the initial predictor, creating a new revised model (Model 1R, see Fig. 4 for more detail). This new path model would allow us to see two pathways of influence towards the presence of bonding difficulties at 8 months postpartum. Results showed that, on the one hand, the birth experience would act as a risk factor predicting the subsequent appearance of P-PTSS, in response to which the mother would use emotional regulation strategies based on the use of brooding, which in turn would trigger depressive symptomatology, ultimately impacting on the quality of bonding. For P-PTSS to impact the quality of bonding, brooding (a maladaptive emotion regulation strategy associated with P-PTSS) and postpartum depression symptomatology (comorbid to P-PTSS and usually caused by this type of brooding ruminative response style) must be considered as potential intervening meditators. Our analyses empirically confirmed these paths and are discussed into detail below. Moreover, in the final model, no direct effect of a negative birth experience on bonding difficulties was found, and it only indirectly influenced it through the above-mentioned path. This suggests that women may have a more difficult or potentially traumatic birth, but this would not necessarily affect her ability to bond with her baby. A negative birth experience would only impact the bond only if postpartum PTSD symptoms is subsequently developed. Thus, it would not be the experience itself but the traumatic interpretation and processing of that experience what would influence the appearance of bonding difficulties.

As suggested by other authors, ruminative response styles would be habitually used by women with P-PTSS as a maladaptive regulation strategy of their experienced distress [20, 62]. A higher use of brooding would not promote trauma integration [20] but instead increase the severity of P-PTSS [20, 22], generating long-term negative emotions [20], among others, being associated to postpartum depressive symptoms. For this reason, in line with our results, P-PTSS should be considered a risk factor for developing postpartum depressive symptomatology, as is the case with general PTSS and depressive symptoms [29–30]. The literature indicates that the causal relationship between PTSD and depressive symptomatology would be due to that people with PTSD have a negative view of themselves, others, and the world, which corresponds to the cognitive triad usually promoting risk for the onset and maintenance of depressive symptomatology [28].

Considering the postpartum stage, the presence of P-PTSS is also related to symptoms of postpartum depression, with the prevalence of this symptomatology at two months of postpartum being between 10 and 20% of women and increasing to 12–25% in new mothers [31]. In addition, postpartum depression tends to be preceded by brooding [26] which comprises a higher attention to negative cognitions referred to the cognitive triad (63. In the final supported model in this study, P-PTSS would predict a ruminative style as a maladaptive regulation strategy of distress, mainly shaped by brooding, being then related to higher postpartum depressive symptom levels.

The results also indicate that postpartum depressive symptomatology impacts the quality of mother-child interactions, favoring higher levels of bonding difficulties [32]. In addition, a higher use of brooding itself, which is responsible for facilitating depressive symptomatology, would also partly directly impact the development of poorer quality mother-child bonding [33]. This may be because brooding drains the mother’s cognitive resources, preventing her from having her baby in mind and thus limiting her ability to respond to her newborn’s needs in a responsive, rapid, and congruent manner [33]. Therefore, the occurrence of P-PTSS would play a further specific indirect role in the generation of bonding difficulties through both ruminative mechanisms as well as through higher associated levels of depressive symptomatology in the postpartum period.

Moreover, the presence of bonding difficulties at two months postpartum would predict, by itself, the continuation of these difficulties at eight months postpartum, both directly and through the emergence of brooding and postpartum depression symptoms. As mentioned earlier, the use of brooding, in this case as rumination in response to difficulties a mother may be experiencing in feeling connected to her baby, would generate long-term negative emotions in the mother [20], implicating a higher attention to negative cognitions referred to the cognitive triad [63], which is associated with postpartum depressive symptoms. This, in turn, impacts the quality of mother-child interactions, leading to higher levels of bonding difficulties [32].

Taken together, these results have several implications that should be considered. The results suggest the importance of developing preventive strategies in the postpartum, including identifying mothers with a previous history of depression and reducing avoidable stress during childbirth to reduce the risk of onset of P-PTSS.

In this line, it is essential to develop training programs for health personnel so that they can learn what aspects can turn childbirth into a traumatic event. By doing so, they can identify those mothers with whom they should work to prevent the development of a traumatic interpretation of childbirth and, consequently, avoid the onset of P-PTSS. In addition, programs focusing on screening and intervening to redirect mothers’ ruminative patterns and depressive symptoms in the early postpartum period would be very helpful in reducing their influence in the appearance of bonding disorders [64].

## Limitations and strengths

Although, as commented above, the present results have several interesting implications for promoting mothers’ mental health and reducing bonding difficulties during the postpartum, a series of limitations must be considered. First, regarding the risk factors for P-PTSS development that were studied, it was only possible to collect pre-birth aspects such as the presence of psychopathology, as well as a broader view of the medical quality received by the health personnel and the birth experience, being based on a general self-report of the mother, assessed with a single question designed for the study that did not show sufficient sensitivity. Future studies should use more precise assessments of these variables and consider other potential risk factors informed by previous literature [12, 13, 18].

Furthermore, the diagnostic criteria established for detecting P-PTSS or postpartum depression state that the assessment should be carried out six months after presenting symptoms [65], but in the present study, it was assessed at eight months postpartum. Similarly, the assessment of P-PTSS was carried out using a PTSD scale designed for its use in the general population due to the lack of validated and standardized questionnaires specifically designed for the perinatal population. Moreover, as for the path model testing longitudinal changes in bonding difficulties, although our model considered theoretical sound paths among variables, by design, most variables needed to be assessed within the same time period, as sociodemographic variables. Thus, further intensive longitudinal research might add value by modelling how each of our supported variables predict such longitudinal changes when modelled across different time points. Similarly, these results refer to a specific socio-cultural (Spain) and health (COVID-19 pandemic) context, which, despite their relevance to the study, have not been controlled for, so it is necessary to be able to replicate this research in other contexts.

Despite these limitations, a series of strengths of the study must be highlighted. First, online recruitment, as the one used in this study, provided an opportunity to obtain larger samples of participants than most of previous studies, and to recruit more women who experience postpartum PTSD or depression, which is useful when studying patho-mechanisms of bonding problems. In this study we were able to conduct a longitudinal study at multiple phases of 150 mothers, which is not typically common, and helped to provide reliable and consistent tests of the hypotheses of our study. Further, the study of factors involved in the onset and maintenance of P-PTSS, as well as its comorbidities and subsequent influences, is a very novel adding of this research, as there are numerous studies about PTSD in the general population [20–22], but not in the perinatal population. Specifically, this study has aimed to provide more extensive evidence about individual cognitive processes, such as a ruminative response style, in studying P-PTSS and its relationship with postpartum depression, that are not typically integrated within this line of research. A limited number of studies have previously explored the relationship between these variables [20] and always in the general population. Furthermore, despite what has been stated about the comorbidity between P-PTSS and depressive symptomatology and the difficulties that this comprises to separate the statistical influence of one on each other [66], the specific nature of the instruments used to assess P-PTSS symptomatology and postpartum depression, made it possible to reduce the covariance of both variables at the statistical level and to isolate the effects of one on each other. Finally, this research highlights the importance of studying the presence of psychopathology during the perinatal stage, specifically in moments of great relevance but less studied, such as the childbirth stage. To this end, the longitudinal approach used in this study highlights the importance of the mother’s life and the pregnancy experience for the understanding the development of problems of mother-child bonding.

## Conclusions

The present study investigated risk factors for P-PTSS in the eighth month of postpartum and the possible mechanisms for its contribution on further bonding problems. The results allow to conclude that previous history of depression, depressive symptomatology during pregnancy, presence of medical complications in the mother, and a negative childbirth experience may be risk factors predicting P-PTSS in the postpartum period. This symptomatology, triggered by a negative childbirth experience, could, in turn, lead to difficulties in mother-child bonding. This relationship would be mediated by the presence of brooding and the development of postpartum depression. P-PTSS would predict a brooding coping style in the mother, increasing the likelihood of depressive symptoms. Moreover, mothers who already had bonding issues at two months postpartum, these could persist at eight months postpartum due to the onset of brooding and postpartum depression symptoms.

These findings are highly informative and may serve as a basis for the development of new future longitudinal studies that continue advancing the understanding of these mechanisms of perinatal mental health. Studies aimed at replicating and extending the relationships between these variables will improve knowledge about the risk factors that are present during the mothers’ life history, her pregnancy, and their influence on their childbirth experience, as well as on their subsequent postpartum mental health, and the quality of mother-child bonding.

## Data Availability

the dataset analysed during the current study is available from the corresponding author on reasonable request.
